# Dissecting mammalian immunity through mutation

**DOI:** 10.1038/icb.2014.8

**Published:** 2014-02-11

**Authors:** Owen M Siggs

**Affiliations:** 1 Wellcome Trust Sanger Institute, Hinxton, Cambridge, UK

**Keywords:** genetics, immunity, mutation

## Abstract

Although mutation and natural selection have given rise to our immune system, a well-placed mutation can also cripple it, and within an expanding population we are recognizing more and more cases of single-gene mutations that compromise immunity. These mutations are an ideal tool for understanding human immunology, and there are more ways than ever to measure their physiological effects. There are also more ways to create mutations in the laboratory, and to use these resources to systematically define the function of every gene in our genome. This review focuses on the discovery and creation of mutations in the context of mammalian immunity, with an emphasis on the use of genome-wide chemical and CRISPR/Cas9 mutagenesis to reveal gene function.

## INTRODUCTION

### The origins of immunity

Since the emergence of unicellular life, there have been few selective pressures more abundant than infection. The evolutionary legacy of infection is a diverse antimicrobial arsenal, known collectively as immunity, that can be found in all species from the simplest prokaryotes to the most complex mammals. Bacteria and archaea, for example, evolved restriction enzymes and CRISPR/Cas (clustered, regularly interspaced short palindromic repeat/CRISPR-associated) systems to combat both phage infection and plasmid conjugation.^1^ Similarly, many antibiotics used in the clinic today are products of prokaryotic competition.

Antibiotics also evolved in eukaryotic organisms, as did a system of sequence-specific RNA silencing. Yet, with the many benefits of eukaryotic multicellularity came vulnerability to new classes of microbes, and therefore a need for new forms of immunity. Receptors for microbial products emerged, exemplified by the Toll protein in *Drosophila*, that in some species has expanded to families of hundreds of paralogs.^2^ Additional microbial molecules were recognized by other receptor families, including the NOD-like, RIG-I-like and C-type lectin receptors.^3^

The crowning achievement of eukaryotic immunity arose in vertebrates not once but twice,^4^ with an anticipatory system of somatically recombined and hypermutated antigen receptors. Not only did this system have the capacity to detect any pathogen that already existed, it had the bandwidth to preempt any pathogen that *would* exist, ever. Although combinatorial diversity created the hazard of self-reactivity, the evolutionary advantage afforded by it was simply too great to be ignored, and mechanisms of self-tolerance emerged as a result. Combinatorial immunity has been refined even further since, with unique structural adaptations to reach even more microbial epitopes.^5, 6^

### Human immunity and the shadows of selection

Just as microbial infection acts as a selective pressure against the host, host immunity exerts powerful selective pressure against the microbe. As quickly as new forms of immunity have emerged, rapid microbial proliferation ensures that they quickly develop ways to avoid them. This perpetual struggle between pathogen and host is reflected by our recent evolutionary history that reveals that immune genes continue to be the most strongly selected elements in our genome.^7, 8^

More recent signatures of selection can be found in human populations with endemic infectious diseases. One classic example is the high prevalence of null mutations in the Duffy antigen gene (*DARC*) in West Africa that confer resistance to the malarial parasite *Plasmodium vivax*. Other positively selected variants have a less obvious origin: the *CCR5Δ32* allele bestows resistance to the modern human pathogen HIV, yet was most likely selected by a more ancient microbe. Certain variants provide such a crucial advantage that they eventually reach fixation in a population because of a selective sweep, and there is evidence that several immune genes fall into this category. A series of parallel selective sweeps is, after all, what separates one species from another, and among other things explains why mice do not become sick after HIV inoculation, or why fruit bats carry Ebola virus without developing hemorrhagic fever.

Other variants with much smaller effects have been uncovered by genome-wide association studies, some of which associate with susceptibility or resistance to infectious disease.^9^ Similar methods have revealed risk variants for autoimmune and inflammatory diseases,^10^ the persistence of which may be a testament to their antimicrobial benefit. A key illustration of this is that loss-of-function variants of *IFIH1* (encoding the microbial RNA sensor MDA5) are associated with resistance to type I diabetes, whereas the more common, functional alleles confer susceptibility.^11^

Although improved hygiene, vaccination and antibiotic use have dramatically reduced the burden of infectious disease over the past 200 years, it remains a powerful selective agent with ∼25% of people ultimately dying from it,^12^ many of whom are young. New pathogens continue to cross from animals into humans, and the pathogens that were once subdued by antimicrobial drugs are rapidly developing resistance. At the same time, the human population is undergoing explosive growth, with new single-nucleotide variants emerging at a rate of ∼1.2 × 10^−8^ per generation.^13^ This is estimated to introduce some 10^11^ *de novo* variants per generation,^14^ but how do we determine which of these variants affect immunity?

### Contemporary experiments of nature

By recent conservative estimates, each human genome carries ∼300 variants that affect protein function.^15^ More than 86% of these are thought to have arisen within the past 10 000 years and therefore have low population frequencies (<5%),^16^ yet because of rapid population growth, most have remained in a heterozygous state and hence have not been subjected to purifying selection.

Nevertheless, in some cases these variants can still cause inherited disease. Some may affect haploinsufficient genes, such as variants in *RPSA* that cause autosomal dominant congenital asplenia.^17^ Other variants act in a dominant manner, such as *PLCG2* mutations in cold-induced urticaria,^18^ or *PIK3CD* variants in a subset of primary immunodeficient patients.^19, 20^ The remainder are either X-linked (such as variants of the T-cell magnesium transporter gene *MAGT1*),^21^ or disrupted at both alleles via consanguinity or compound heterozygosity (homozygous *IRF8* mutations in mycobacterial susceptibility being an example of the former^22^). These experiments of nature have taught us a great deal about immunity, not only in humans but also in animals. Some of the biggest conceptual breakthroughs in the past 20 years of immunology have emerged from the study of spontaneous mutations, from the understanding of microbial sensing (*Tlr4*) and dominant tolerance (*Foxp3*) in mouse mutants,^23, 24^ to the discovery of multiple variants causing monogenic autoimmunity, immunodeficiency or sterile inflammatory disease in humans.

### Making sense of nature's experiments

Revealing the genetic basis of an inherited immune disorder lays a foundation for hypothesis and further experimentation. Fortunately, many human immune cells are accessible enough to test these hypotheses, and in many cases a patient blood sample is the perfect starting point. Occasionally, the critical cell type may be unavailable or inaccessible, for example, in *TLR3*-associated viral encephalitis, where the central nervous system is suspected to be central to pathogenesis. An emerging solution is the use of patient-derived induced pluripotent cells that can be differentiated into the appropriate cell lineage and studied *in vitro* alongside controls.^25^ An even better alternative is to use isogenic human induced pluripotent cells, where experimental and control cell lines differ by only a single genetic variant. Sequence-specific genome editing tools have been invaluable in this context, with the CRISPR/Cas9 platform emerging as the most versatile.

For many purposes these *in vitro* platforms will be sufficient, yet for many physiological phenomena they are not. Model organisms, and the mouse in particular, provide the most powerful means available to study mammalian physiology, offering an environmentally and genetically controlled platform for the study of cellular interactions within the context of a whole organism. For the 99% of human genes that have a direct counterpart,^7^ the mouse has been the dominant model for understanding their function. The tools available for engineering the mouse genome are without equal, with transgenesis, homologous recombination and sequence-specific nucleases used to create a spectrum of strains that have become a cornerstone of modern immunology. The speed and ease with which the CRISPR/Cas9 platform can create germline mutations makes the mouse an even more accessible platform for examining the effects of human genetic variation.^26^

Laboratory mice are nevertheless an imperfect model of human immune disease,^27^ as might be expected after 65 million years of genetic divergence. Although invaluable experimental interpretation and consistency, the widespread use of inbred strains of mice (particularly C57BL/6) fails to capture the genetic and phenotypic diversity of outbred human populations. A more suitable experimental model of immunological disease is not immediately obvious, although it may one day involve some combination of genetically humanized mice with transplanted human cells.

## SYSTEMATICALLY CONNECTING GENOTYPE TO IMMUNE PHENOTYPE

Of the ∼20 000 protein coding genes and several thousand noncoding RNA genes in our genome, we know remarkably little about the normal function of most of them. At the same time, the catalog of human genetic variation is expanding exponentially, yet our understanding of its biological significance is languishing in its wake. The vast majority of our understanding of mammalian gene function has come from single-gene mutations in mice, with upwards of 7000 genes having at least one annotated phenotype.^28^ Mendelian human diseases follow closely behind, with approximately half as many individual genes linked to an inherited phenotype.^29^ Several efforts are now underway to systematically dissect the biological function of every human gene, and the relative strengths of each will be discussed below.

### Systematic clinical phenotyping and genome sequencing

The most relevant way to understand the function of a human gene is to study mutations in humans themselves. Given the germline mutation rate and a population of >7 billion, it is reasonable to expect that multiple null alleles of every human gene already exist, some of which may be in a compound heterozygous or homozygous state. Our immune systems are also constantly challenged by infection, by immunization and (in some cases) by immune-modifying drugs. Together with clinicians, we are constantly monitoring the function of our immune systems, and those individuals with the most severe defects are quickly noticed. Many of these severe immune disorders are caused by mutations in a single gene, with more than 200 such genes now defined.^30^ This ability to connect individual human genes to immune function is immensely valuable, and the advent of massively parallel sequencing has meant that dozens more are discovered each year.

Functional variants not associated with clinical disease will be more difficult to identify. Some individuals may not respond to a given vaccine, for example, yet remain healthy because of herd immunity. Similarly, the highest responders will also be healthy, but may harbor valuable genetic clues for enhancing immunity in those with less capable immune systems. Identifying these individuals will require robust and comprehensive methods of immune phenotyping,^31^ and many hundreds or thousands of cases and controls to extract reliable genetic associations.

### Targeted germline mutagenesis and phenotyping

A definitive way of determining mammalian gene function has been the creation of targeted gene knockouts in mice. Candidate genes are typically selected with an explicit hypothesis in mind: one that is often based upon previous *in vitro* experiments, homology to known immune genes or by comparative gene expression analysis. The creation and phenotyping of mutant mice is an efficient way to understand the function of families of genes, but usually ignores genes that are not suspected by prior hypothesis, or ignores phenotypes that are beyond a laboratory's sphere of expertise.

**Figure 1:**
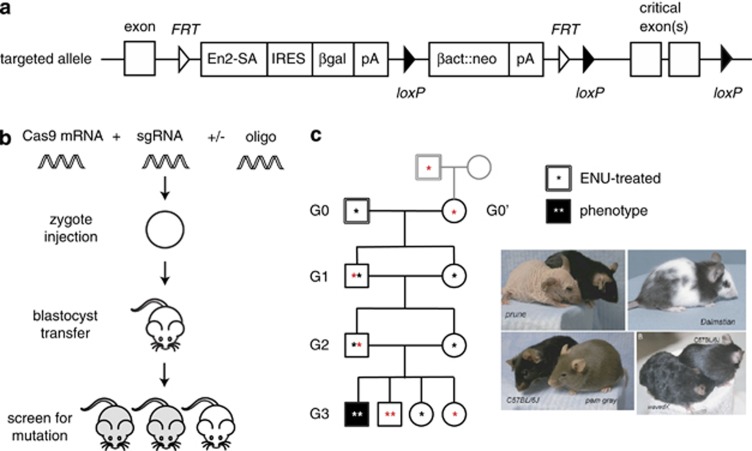
Contemporary methods for mouse germline mutagenesis. (**a**) Standard design of a *lacZ*-tagged conditional allele generated by the European Conditional Mouse Mutatgenesis (EUCOMM) targeting pipeline.^32^ In its default state, the allele is inactivated by splicing upstream exons to a splice acceptor in the targeting cassette. Treatment with Flp recombinase can delete the selection cassette to create a *loxP*-flanked conditional allele. βact::neo, β-actin promoter-driven neomycin resistance cassette; βgal, β-galactosidase; En2-SA, mouse En2 splice acceptor; *FRT*, *FRT* recombination site; IRES, internal ribosome entry site; *loxP*, *loxP* site; pA, SV40 polyadenylation signal. (**b**) CRISPR/Cas9-mediated generation of targeted mutations. Mammalian codon-optimized Cas9 mRNA and target-specific single-guide RNA(s) are injected into zygotes, leading to the creation of indels as a consequence of double-stranded break repair^26^ (see text for more detail). Multiple alleles and multiple genes may be targeted simultaneously. DNA oligos or vectors with target homology may also be coinjected, allowing the introduction of point mutations or exogenous DNA by homology-mediated repair.^65^ (**c**) Pedigree structure for the generation of ENU-induced homozygous germline mutations. Male C57BL/6J pedigree founders are treated with ENU, and bred with either a wild-type female, or with the female offspring of an ENU-mutagenized male (G0′). After two successive generations of brother–sister mating, recessive mutations can be brought to homozygosity, and both recessive and dominant phenotypes may be identified in third-generation (G3) mice. Examples of visible phenotypes with recessive (prune (*Hr*), pam gray (*Hps3*), wavedX (*Adam17*)) or dominant (Dalmatian (*Sox10*)) inheritance are shown.

Previously created by specialist laboratories on a case-by-case basis, gene-targeted stem cells can now be ordered directly from large public repositories^32^ (Figure 1a). These archives include alleles made by both random gene trap insertions and by targeted homologous recombination (and, inevitably, by CRISPR/Cas9), and are on track to create null alleles of every gene in the mouse genome. The bottleneck now lies in the conversion of these embryonic stem (ES) cell clones into mutant mice: blastocyst injections and at least two generations of breeding are required to obtain the desired homozygous mutants.

A comparatively rapid alternative to targeting in mouse embryonic stem cells is to create mutations directly in zygotes. Sequence-specific nucleases (including zinc-finger nucleases and transactivator-like effector nucleases) have been used to generate biallelic null mutations within a single generation. Yet, neither zinc-finger nucleases nor transactivator-like effector nucleases are as simple to engineer as the CRISPR/Cas9 system that was recently adapted from *Streptococcus pyogenes* for genome modification in mammals.^33, 34^ Coinjection of mRNA for the Cas9 endonuclease and a single-guide RNA (sgRNA, containing 20 bp of complementarity to the target) is sufficient to obtain high frequencies of biallelic null mutations in the target of choice, with few off-target effects and few restrictions on the location of the sgRNA target^26^ (Figure 1b).

Along with archives of targeted mouse mutants, the establishment of high-throughput phenotyping pipelines has meant that diverse phenotypes can be explored systematically in a single facility, rather than sequentially over many years in several different labs. This has already led to some unexpected discoveries, with new phenotypes detected for 56 of the first 250 genes analyzed.^28^ Many of these genes had been examined by specialist laboratories in the past, but certain physiological functions had never been suspected and thus were never examined (or reported). Although it requires an enormous amount of coordinated effort and resources, broad phenotyping can nonetheless expose entirely unexpected gene functions, in addition to eliminating others. In the domain of immunity, current assays include complete blood count, lymphocyte flow cytometry and infection with *Salmonella typhimurium.*^28^ More specialized immune pipelines have also been launched, including more comprehensive flow cytometric analysis of primary and secondary lymphoid organs, detection of autoreactive antibodies and the challenge of intestinal integrity (A Hayday *et al.*, personal communication). Far from exhaustive, these pipelines are designed to provide a standardized public resource of broad phenotypic data, with all mutant lines and data made publicly available for further analysis. The majority of these alleles can also be converted to a conditional state,^32^ allowing investigators to study genes in a cell- and tissue-specific manner: an especially useful feature for the ∼30% of genes that are homozygous lethal when mutated.^28^

### Random germline mutagenesis

A key limitation of large-scale phenotyping efforts is the depth of phenomena that can be tested. Immunology is awash with phenomena: specialized subsets of leukocytes are constantly being described, and new pathways for microbial recognition continue to emerge. There are simply too many to monitor in a single pipeline, and therefore many important phenotypes will inevitably be missed. Almost a third of null alleles are also embryonic lethal in homozygous form, yet missense alleles of many of the same genes can be viable (and sometimes even associated with disease^35^).

Random germline mutagenesis provides a way to circumvent some of these issues. Radiation, chemicals, retroviruses and transposons can all create germline mutations at random *in vivo*, although the most widely used mouse germline mutagen is the alkylating agent *N*-ethyl-*N*-nitrosourea (ENU). At an optimal dose, ENU creates single-nucleotide variants in mouse spermatogonial stem cells every 600 000 bp;^36^ ∼500 times higher than the *de novo* missense mutation rate in humans. Optimally treated male founders transmit ∼60 genetic variants that alter the proteins that they encode, and with an appropriate breeding scheme (Figure 1c) ∼6 such mutations can be examined in a homozygous state in every third-generation (G3) mouse, with an additional 39 that are heterozygous.^37^ A pipeline such as this can be established within a year and requires no technical expertise beyond intraperitoneal injection and mouse husbandry, and comes at a substantially lower per-gene cost than creating the equivalent number of targeted mutations. Unlike a well-designed knockout allele, not all ENU mutations will disrupt protein function, although current estimates suggest that ∼15% of them do.^38^ Some of these variants create null alleles, others create a partial loss of function (where full loss of function is often lethal) and some can even lead to gains of function. Even where a knockout allele has been studied in depth, the phenotypes resulting from missense alleles can sometimes also have unexpected effects,^39^ and therefore reveal even more about the proteins and pathways they affect.

Phenotyping assays applied to an ENU screen should have high specificity and sensitivity, as in many cases only a single homozygous mutant (or a few heterozygous mutants) will be phenotyped (as opposed to several in the case of targeted mutagenesis pipelines). The most effective immunological screens to date have focused on cellular phenotypes measured by flow cytometry^40^ (Table 1), complete blood count^41^ or Toll-like receptor-induced cytokine production,^42, 43^ although others measuring systemic phenotypes such as antigen-specific antibody production,^44^ intestinal integrity^45^ and susceptibility to infection^46, 47^ have also been successful. A low false positive frequency is crucial for saving time and resources, and high sensitivity is important to ensure that genuine phenotypic mutations are not missed.

Once a phenotype is identified, whole-genome or whole-exome sequencing can quickly establish a list of potential causative mutations. Sequencing only a single affected mouse will reveal dozens of variants, and therefore each variant should be genotyped across multiple affected individuals to test for phenotypic concordance. This often points to a probable causative variant, although transgenesis or allelism testing (or some other form of complementation) is necessary to formally assign cause and effect. The vast majority of causative variants are those that change protein coding sense (with very few exceptions^48^), making exome sequencing a popular platform for ENU mutation discovery. In comparison, an advantage of genome sequencing (when performed across multiple samples) is that it can also define the chromosomal boundaries within which the causative mutation must lie^36^—a valuable asset when an obvious candidate is not found on the first pass.

### Haploid cell mutagenesis

Not all immunological phenotypes require whole organisms to study, and indeed many can be more efficiently studied in individual cells. Although biallelic mutations have been rather difficult to create in diploid mammalian cells, random mutagenesis of cancer cell lines has nonetheless revealed several key components of T-cell receptor, interferon and nuclear factor-κB signaling pathways.^49, 50, 51^

The development of haploid human cell lines (namely, the KBM-7 chronic myelogenous leukemia line) has allowed the hurdle of diploidy to be bypassed, and the advent of massively parallel sequencing means that mutations can be found with increasing ease. This has led to a resurgence of interest in cellular mutagenesis,^52^ and millions of null mutations can now be screened simultaneously within pools of cells, for example, by selecting for resistance to viruses or bacterial toxins^52, 53, 54^ or by selecting for the absence of key cell surface molecules.^55, 56^

Haploid mouse ES cells are potentially even more versatile,^57^ given their capacity to differentiate into a wide variety of lineages. Although not yet as stable as their immortalized counterparts, ES cell haploidy can be maintained by regular cell sorting for haploid DNA content. Mutagenizing cells immediately after enrichment ensures that mutations will persist in either a hemizygous (if haploid) or homozygous (if diploid) state, and can therefore be used directly for recessive screens.^57, 58^ By combining this approach with directed differentiation of ES cells into other lineages, the spectrum of available phenotypes expands even further. Human haploid ES cells could also serve as a valuable *in vitro* platform, and although they are yet to be reported, recent success with macaque embryos suggest that human haploid ES cell lines may not be far away.^59^

### CRISPR/Cas9 cellular mutagenesis

Perhaps the most versatile mutagenesis platform of all has emerged with the development of mammalian CRISPR/Cas9 mutagenesis. Recently adapted from the bacterium *S. pyogenes*, the CRISPR/Cas9 system requires coordinated expression of two components: a mammalian codon-optimized Cas9 endonuclease, and a chimeric sgRNA consisting of CRISPR RNA (crRNA) and *trans*-acting crRNA elements.^60^ Each crRNA contains a 20-bp guide sequence that directs the sgRNA/Cas9 complex to its complementary DNA target, and repair of the ensuing double-stranded break ultimately results in small deletion (or insertion) mutations at the target site. The sgRNA/Cas9 complex will continue to cut its target until disrupted by an indel, and therefore diploidy and polyploidy present no particular hurdle.

**Figure 2:**
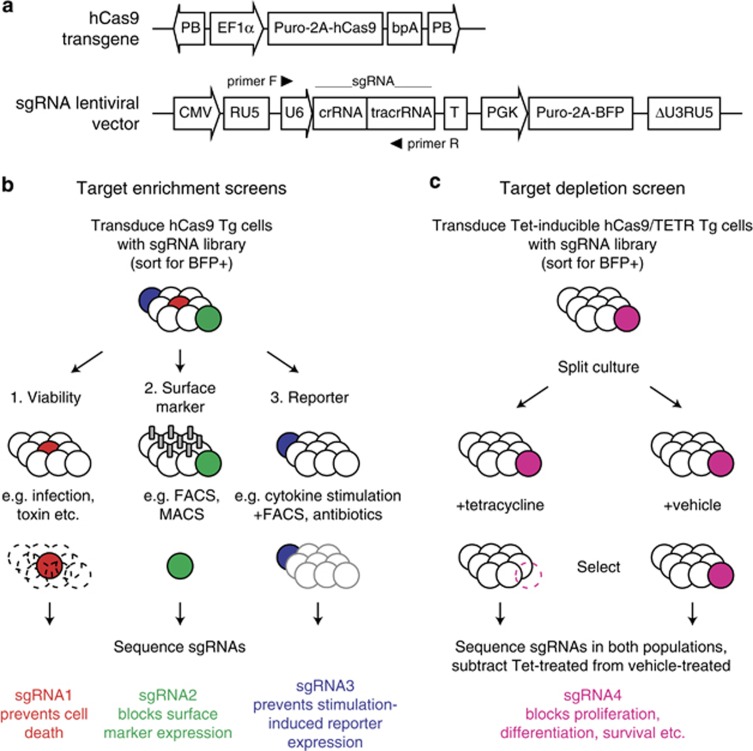
Genome-wide CRISPR/Cas9 mutagenesis. (**a**) Design of hCas9 expression vector and sgRNA lentiviral vector for genome-wide CRISPR/Cas9 mutagenesis screens (adapted from Koike-Yusa *et al.*^61^). CMV, CMV promoter; EF1α, elongation factor-1α promoter; PB, *piggyBac* repeats; PGK, mouse *Pgk1* promoter; Puro-2A-hCas9, puromycin resistance cassette and humanized Cas9 cDNA separated by the T2A self-cleaving peptide; Puro-2A-BFP, puromycin resistance cassette and blue fluorescent protein cDNA separated by the T2A self-cleaving peptide; RU5, 5′ long terminal repeat; sgRNA, single-guide RNA; T, U6 terminator; tracrRNA, *trans*-activated crRNA; U6, U6 RNA polymerase III promoter; ΔU3RU5, RU5 long terminal repeat lacking U3 region. (**b**) Target enrichment screens, which enrich for cells with the mutation of interest, can be performed with a wide variety of selective agents. Similar screens have successfully been performed in haploid cell lines using transposon-mediated mutagenesis.^52, 55, 56, 57, 66^ In the context of genome-wide CRISPR/Cas mutagenesis, an approach validated by Koike-Yusa *et al.*^61^ begins with the transduction of hCas9-expressing cells with a lentiviral genome-wide library of sgRNAs. Transduced cells can be selected by sorting for BFP+ cells, in which sgRNA/Cas9-mediated double-stranded breaks lead to the introduction of indel mutations. These transduced cells can then be subjected to an array of selective pressures, including drug- or pathogen-induced cell death, fluorescence or magnetic cell sorting, or the induction of a stimulus-responsive selection marker. sgRNA sequences can then be sequenced in the remaining cells, and used to identify target genes that are critical for the phenomenon of interest. (**c**) Target depletion screens, also known as ‘dropout' screens, use subtractive methods to identify genes critical for a given phenomenon. Cells are first transduced with a drug-inducible hCas9 construct (along with the TetR transcriptional repressor, in the case of a tetracycline-inducible system), then stable lines are transduced with a genome-wide library of sgRNAs. The pool of BFP+ cells is then split into two, with hCas9 expression induced in one but not the other. hCas9 expression leads to the creation of sgRNA-directed indels, a subset of which will be detrimental to the survival of the cell (or its proliferation or differentiation). Following selection for the phenotype of interest (for example, survival), sgRNA sequences are amplified from the remaining induced and noninduced populations and sequenced. Those sgRNAs that are present in the noninduced population, but absent from the induced population, must therefore be suspected to target a critical gene. These screens are conceptually similar to pooled short hairpin RNA (shRNA) screens, in which barcoded inducible shRNAs are transduced into target cells before selection.^64^

sgRNA/Cas9 target sites can be selected across the genome: the only requirement is that they precede an NGG trinucleotide sequence (also known as the protospacer adjacent motif). Different Cas proteins have different protospacer adjacent motif requirements, although Cas9 is currently the dominant nuclease used in mammalian cells and can potentially target ∼40% of all human exons.^34^ However, not all crRNAs are equally effective (nor are they all specific), and hence individual genes should ideally be targeted independently with multiple sgRNAs. As the sgRNA guide sequence itself is only 20 bp, genome-wide libraries can be readily synthesized and cloned into transducible vectors. Lentiviral vectors are especially appealing for this purpose, given their ability to transduce a wide array of cell types from many different species (Figure 2a).

As with all cellular screens, CRISPR/Cas9 screens can be performed clonally with individual vectors, yet the most efficient screens are those that can be done with a pooled vector library on large populations of cells.^61, 62, 63^ An inherent benefit of pooled sgRNA screens is that each sgRNA sequence doubles as a molecular barcode, such that the depletion or enrichment of individual sgRNAs can easily be linked back to the gene it targets by deep sequencing. This makes it possible to perform saturation mutagenesis screens for essentially any cell-intrinsic phenotype, so long as a reliable means of phenotypic selection is available (Figures 2b and c). A similar approach is commonly used with lentiviral short hairpin RNA libraries,^64^ and the same screening principle can potentially be applied to any transducible cell from any organism: a published genome sequence being the only prerequisite for library design.

There are dozens of immunological phenomena that might be subjected to a genome-wide cellular screen. For instance, how does a pathogen infect its target cell, and how does that cell react? How does a hematopoietic stem cell give rise to a diverse range of lineages, and what allows a malignant lymphocyte to escape these boundaries of proliferation? These questions and many others may all be addressed with cellular CRISPR/Cas9 mutagenesis.

## CONCLUSION

Mutation has made our immune system what it is today, but a single mutation can also reveal its most obscure secrets. Billions of new genetic variants are entering the human gene pool in every generation, and those that compromise immunity will inevitably end up in the clinic. In many cases, finding the causative mutation is now easier than ever before, and there are new tools to measure the immunological effects of genetic variation in healthy individuals. It is also easier to create mutations in an experimental setting, be they in a mouse or a human cell, and public resources of mutant cells and organisms are more accessible than ever. Applying these resources to systematically understand immunity requires coordinated effort, but genome-wide mutagenesis platforms are becoming accessible even to the modestly funded laboratory.

The field of immunology is already faced with an abundance of questions, and single-gene mutations in humans, animals or cells have been some of the very best tools to answer them. But new questions are emerging at every turn: only 20 years ago were we oblivious to the molecular basis of microbial recognition, regulatory T cells and innate lymphoid cells. The acquired immunodeficiency and severe acute respiratory syndromes (and the pathogens that cause them) are also relatively new. For all of these phenomena, and indeed for those that might emerge next, there is much to be gained from the continued application of mutation.

## Figures and Tables

**Table 1:** Examples of immunological gene function revealed by flow cytometric screening of mouse pedigrees with ENU-induced germline mutations

*Gene*	*Affected lineage*	*Human Mendelian disease variants?*	*Reference*
*Atp11c*	B		^67, 68^
*Card11*	T, B	Yes	^69^
*Cd83*	T, DC		^70^
*Coro1a*	T	Yes	^71^
*Fnip1*	B		^72^
*Gfi1*	Neu	Yes	^73^
*Gon4l*	B		^74^
*Hnrnpll*	T		^75^
*Ikzf1*	T, B		^76^
*Il7*	T, B		^77, 78^
*Itgb2*	NK	Yes	^79^
*Itpkb*	T		^80^
*Lig4*	T, B	Yes	^81^
*Lyn*	B		^82^
*Myb*	LT-HSC		^83^
*Ncaph2*	T		^84^
*Nckap1l*	T, B		^85^
*Nfkb2*	B		^86^
*Ptprc*	T	Yes	^87^
*Rltpr*	T		^88^
*Sppl2a*	B, DC		^89, 90^
*Tap1*	T	Yes	^91^
*Tap2*	T	Yes	^91^
*Tbx21*	NK		^92^
*Themis*	T		^93, 94^
*Zap70*	T	Yes	^39, 95^
*Zbtb1*	T, B, NK		^96^
*Zbtb7b*	T, NKT		^97^

Abbreviations: B, B cell; DC, dendritic cell; ENU, *N*-ethyl-*N*-nitrosourea; LT-HSC, long-term hematopoietic stem cell; Neu, neutrophil; NK, natural killer cell; T, T cell.
